# Biomechanical Analysis of Human Gait When Changing Velocity and Carried Loads: Simulation Study with OpenSim

**DOI:** 10.3390/biology13050321

**Published:** 2024-05-04

**Authors:** Cristina Brambilla, Giulia Beltrame, Giorgia Marino, Valentina Lanzani, Roberto Gatti, Nicola Portinaro, Lorenzo Molinari Tosatti, Alessandro Scano

**Affiliations:** 1 Institute of Intelligent Industrial Systems and Technologies for Advanced Manufacturing (STIIMA), Italian Council of National Research (CNR), 20133 Milan, Italy; cristina.brambilla@stiima.cnr.it (C.B.); valentina.lanzani@stiima.cnr.it (V.L.); lorenzo.molinaritosatti@stiima.cnr.it (L.M.T.); 2 Residency Program in Orthopedics and Traumatology, Universitá degli Studi di Milano, 20122 Milan, Italy; beltramegiulia.gb@gmail.com (G.B.); nicola.portinaro@humanitas.it (N.P.); 3 Physiotherapy Unit, IRCCS Humanitas Research Hospital, Rozzano, 20098 Milan, Italy; giorgia.marino@humanitas.it (G.M.); roberto.gatti@hunimed.eu (R.G.); 4 Department of Biomedical Sciences, Humanitas University, Pieve Emanuele, 20072 Milan, Italy; 5 Department of Pediatric Surgery, Fondazione IRCCS Ca’ Granda, Ospedale Maggiore Policlinico, 20122 Milan, Italy

**Keywords:** biomechanics, OpenSim, musculoskeletal model, speed, load, gait

## Abstract

**Simple Summary:**

Simulation approaches are widely used in biomechanics. One of their main applications is to compute inverse dynamics to estimate joint torques and muscle activations, especially in conditions hard to reproduce experimentally. In this work, we used the OpenSim software to investigate the effects on joint and muscular biomechanics of the lower limb during gait when gait velocity changes and different loads are carried by the subject, starting from a dataset of gait data. We found that biomechanics were influenced by both speed and load. Our results expand the previous literature by providing comprehensive data in multiple conditions that cannot be easily tested in experimental trials. This study could be useful for applications in many areas, such as rehabilitation, orthopedics, medical care, and sports.

**Abstract:**

Walking is one of the main activities of daily life and gait analysis can provide crucial data for the computation of biomechanics in many fields. In multiple applications, having reference data that include a variety of gait conditions could be useful for assessing walking performance. However, limited extensive reference data are available as many conditions cannot be easily tested experimentally. For this reason, a musculoskeletal model in OpenSim coupled with gait data (at seven different velocities) was used to simulate seven carried loads and all the combinations between the two parameters. The effects on lower limb biomechanics were measured with torque, power, and mechanical work. The results demonstrated that biomechanics was influenced by both speed and load. Our results expand the previous literature: in the majority of previous work, only a subset of the presented conditions was investigated. Moreover, our simulation approach provides comprehensive data that could be useful for applications in many areas, such as rehabilitation, orthopedics, medical care, and sports.

## 1. Introduction

Walking is one of the main activities of daily life performed by humans and it is considered a key factor for functional independence [1]. Walking activity allows us to move in different environments and it is achieved by coordinated movements of body segments, interacting with internal and external forces [2]. Gait is a cyclic activity, made of a repetitive series of limb movements and it is characterized by specific patterns and two main, sequential phases: stance and swing [3]. Abnormal patterns are associated with neurological, muscular, or skeletal pathologies [1]. An instrumented gait analysis allows us to record the walking activity and to assess biomechanical parameters, providing comprehensive data useful in a wide variety of contexts [4]. In multiple applications, it is necessary to have reference values for biomechanical parameters and to know how these values change in relation to alterations of “standard” gait conditions. In the medical context, the assessment of the torques acting on joints can be used for the diagnosis and treatment of patients, for the design of gait assistive devices, and for the evaluation of the treatment effects [2]. Moreover, gait reference values may be useful for assessing the effects induced with lower limb exoskeletons that are used in rehabilitation, by comparing gait biomechanics with the exoskeleton to the biomechanics of free walking [5]. In orthopedic surgery, gait analysis is performed to evaluate pathological conditions and to make decisions on the intervention and to assess the outcome of the surgery [6]. In sports, a biomechanical analysis is needed to assess movement techniques of athletes [7] and to improve their performance and movement efficiency, by maximizing power output or reducing the work required for a task [8]. In the industrial scenario, biomechanical analysis is used for the evaluation of performance and ergonomics and to ensure workers’ safety [9,10]. Furthermore, in the human–robot interaction, biomechanics can be useful for the evaluation of the interaction with other devices, such as exoskeletons for weight support or collaborative robots that can modify gait and lower limb movements of the worker [11].

However, no extensive data are present in the literature that comprehend multiple conditions, especially including high variability in speed and loads, that are typical parameters that may alter the biomechanics of human gait. It was shown that the ground reaction forces (GRFs) increase proportionally to the increase in the carried load, and this reflects in higher joint torques and muscle activations [12,13]. In the literature, the effects of walking speed on kinematics and biomechanics have been studied with experimental trials but only limited ranges of velocity have been assessed, finding that torque and power of all joints increase with speed [14,15]. Similarly, muscle activations increase with walking speed in both experimental [14] and simulated studies [16]. Few studies analyzed the effects of carried load on the lower limb biomechanics during gait and the combination with speed. Middleton et al. analyzed the effects of carrying loads on biomechanics of men and women, finding that all joint kinetics increase with load [17]. Lenton et al. investigated the effects of two loads and two walking speeds on lower limb biomechanics, finding that it is affected by both load and speed, especially for the hip joint [18]. However, most of the studies suffer from a limited number of experimental conditions.

In order to include different gait conditions, when changing speed or adding loads, we used data from a publicly available dataset [14]. The simulation approach allowed us to include eight different carried weights and combine velocities and carried loads. The simulation also included the computation of dynamic parameters that cannot be easily investigated in experimental scenarios, due to the high number of trials, or the need of dressing the subjects with a high number of sensors, or with heavy loads. OpenSim was used for the simulations since it is a widespread used tool, especially for a gait analysis. Previous studies used OpenSim for the evaluation of the effects on kinematics while wearing shoes [19], for the estimation of muscle force during walking [20,21], and for assessing joint and muscle forces in slow and fast gait [22].

Thus, seven speed values (from real data) and seven load conditions (simulated) were tested in the simulation environment of OpenSim, and all the combinations of the two parameters. The aim of this study is to analyze the effects of gait speed and carried load on the biomechanics of the lower limbs and to provide an extensive comprehensive dataset. 

## 2. Materials and Methods

**Figure 1:**
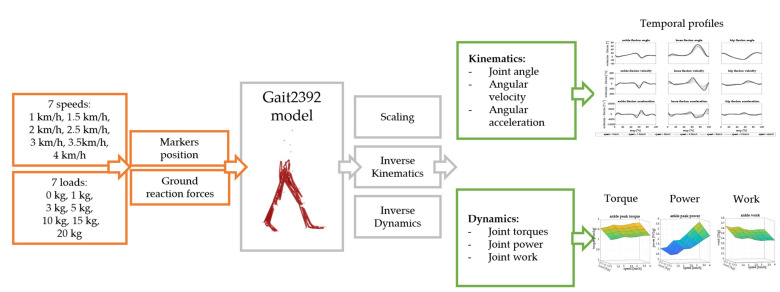
The workflow of the study. Marker positions were imported from real data. Ground reaction forces from real data were scaled to simulate the effect of seven carried loads and they were given as input to the musculoskeletal model. Simulations were performed with the Gait2392 model in OpenSim. Scaling, inverse kinematics, and inverse dynamics were performed and kinematic and dynamic parameters were analyzed.

The scheme of the work is shown in Figure 1.

### 2.1. OpenSim Model

Musculoskeletal simulations were performed in OpenSim v4.4 [23], using the available 3D Gait2392 model [24,25,26] for simulating human gait. This model has 23 degrees of freedom and features 76 muscles of the lower limbs. Experimental data were taken from a publicly available dataset of 15 subjects performing gait at 7 different velocities [14]. A total of 15 subjects (7M, 8F; 23.8 (2.1) years; 1.69 (0.11) m; 66.6 (10.8) kg) were considered for this study. The motion analysis assessment protocol consisted in 24 markers, attached to the body according to the protocol of Moreira et al. [14]. Marker trajectories and ground reaction forces (GRFs) are provided with a sample frequency of 200 Hz. The OpenSim model was scaled to meet each participant’s anthropometry. The inverse kinematics was computed giving the 3D marker trajectories as input. Then, the inverse dynamics were computed with the resulting kinematics and the GRFs. 

### 2.2. Simulations

In order to investigate the effects of gait speed and carried load on the kinetics of the lower limbs, the GRFs were scaled. A variety of speeds and loads were selected to map a high number of biomechanical conditions. The speed was incremented in a reasonable range of gait velocities according to the data from Moreira et al. [14]. The gait speed was changed from 1 km/h to 4 km/h with steps of 0.5 km/h; the carried loads were 0 kg, 1 kg, 3 kg, 5 kg, 10 kg, 15 kg, and 20 kg. The load values included both light and heavy loads that can be related to loads carried in daily life activities. The carried load was an external load concentrated vertical force applied in a point positioned 2cm behind the center of mass in the sagittal plane and 30 cm above the center of mass in the vertical axis to simulate a backpack carriage. 

The vertical and sagittal components of the GRF were scaled according to the load. Both vertical and sagittal components were scaled proportionally to the added load:

$$F_{sag,load}=F_{sag,exp}\cdot \left(1+\frac{load}{weight}\right)$$

$$F_{vert,load}=F_{vert,exp}\cdot \left(1+\frac{load}{weight}\right)$$

where $F_{sag,load}$ and $F_{vert,load}$ are the scaled sagittal and vertical components of the GRFs, $F_{sag,exp}$ and $F_{vert,exp}$ are the experimental sagittal and vertical components of the GRFs, load is the carried load, and *weight* is the weight of the subject.

### 2.3. Outcome Measures

We considered one stride performed with the right limb for the analysis of each subject. The events for segmenting each stride were defined as two consecutive contacts of the right heel with the ground. Therefore, a stride consists in the stance phase (when the right foot is in contact with the ground) and the swing phase (when the right foot is raised and moved forward). Articular angles and joint torques were obtained from the OpenSim model and they were filtered with a 3rd-order Butterworth low-pass filter with a cut-off frequency of 6 Hz. The degrees of freedom considered for the analysis were the ankle flexion, the knee flexion, and the hip flexion. Angular velocity and acceleration for each joint were computed as the first and second derivative of the articular angles, respectively. Joint moments were computed with OpenSim and normalized by the weight of each subject. Joint power was computed as the product between the joint torque and angular velocity, while work was computed as the integral of the joint power in time. As outcome measures, we reported the peak torque, the peak power, and the total mechanical work. These parameters are computed for ankle, knee, and hip joints. 

### 2.4. Statistics

The distributions of the peak torque, peak power, and total mechanical work were tested for normality with the Kolmogorov–Smirnov tests. Then, peak torque, peak power, and mechanical work were analyzed separately. Each parameter was compared across changing speed and load with a 2-way ANOVA test with replicates to investigate the effects of speed and load and their interaction. The level of significance was 0.05. A multiple-comparison was performed to assess the differences across speeds and across loads. Each joint was analyzed separately.

## 3. Results

**Figure 2:**
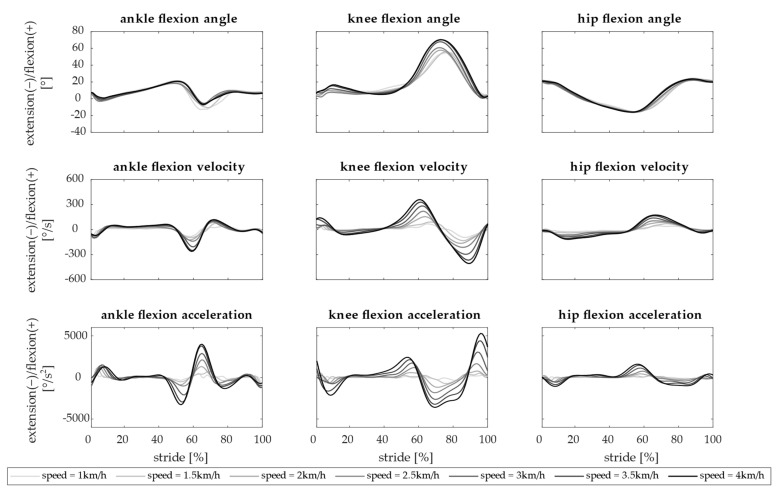
Kinematics of the ankle, knee, and hip. The angle, angular velocity, and acceleration computed for a single stride are reported in rows for each joint (ankle, knee, and hip—in columns). Joint flexion is positive and extension is negative. Each line is the average across 15 subjects. Seven speeds are represented (from 1 km/h in light gray to 4 km/h in black).

The kinematics of ankle, knee, and hip joints averaged across subjects is reported in Figure 2. 

The ankle flexion had the lowest range of motion and reached 20° of flexion during the toe-off, before starting the swing phase, and its profile was repeatable across speeds. The knee angle anticipated and increased the flexion peak, at increasing speeds, and reached a maximum flexion angle of 71° during the swing phase. The hip joint was extended during the stance phase until 15° and its profile slightly changed with speed. Velocities and accelerations were higher when the gait speed increased. 

**Figure 3:**
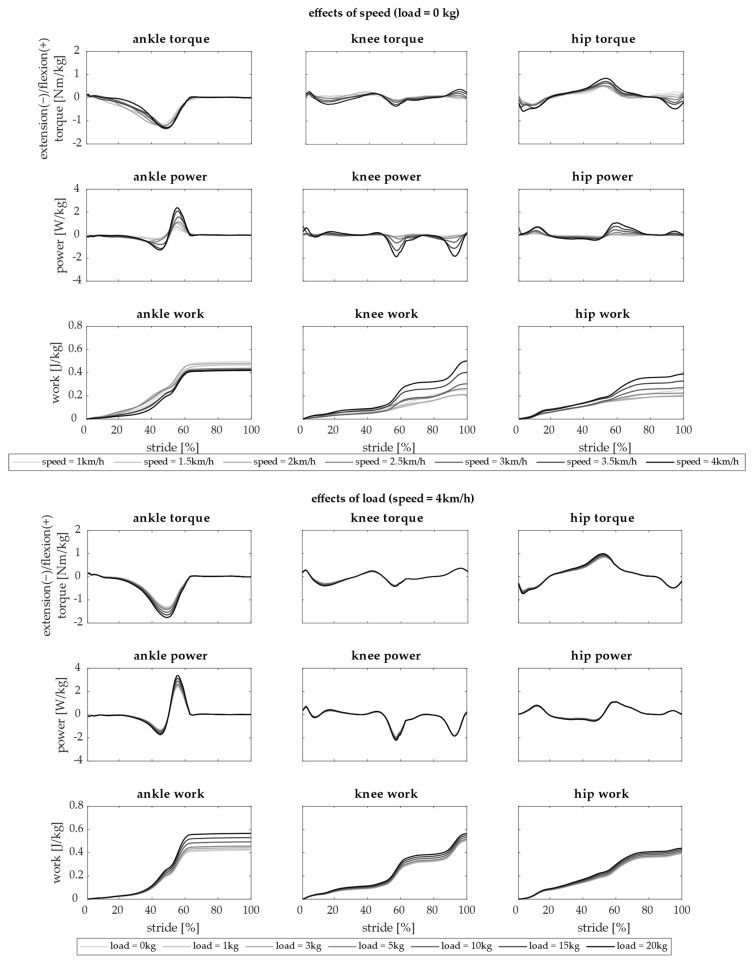
Dynamics of the ankle, knee, and hip. Torque, power, and work computed during a single stride averaged across 15 subjects are reported in rows for each joint (ankle, knee, and hip—in columns). In the upper panel, the effects of changing speed (from 1 km/h in light gray to 4 km/h in black) with load = 0 kg are shown. In the lower panel, the effects of changing load (from 0 kg in light gray to 20 kg in black) at 4 km/h are shown.

An example of how speed and load affect dynamics of ankle, knee, and hip joints is reported in Figure 3. Torque, power, and work are shown when changing gait speed with load = 0 kg in the upper panel and when changing carried load at 4 km/h in the lower panel.

Dynamics of the ankle, knee, and hip were affected by both load and gait speed. In general, gait speed influenced the dynamics more with respect to the added load. Indeed, the differences due to the load were lower than the one due to the speed. Speed increased the peaks of torque and power of all joints and increased the work of the knee and hip. Interestingly, ankle work decreased with increasing speed. Carrying a load, instead, resulted in higher peaks of all the dynamic parameters of the joints. In particular, the ankle was the most affected joint due to load. 

**Figure 4:**
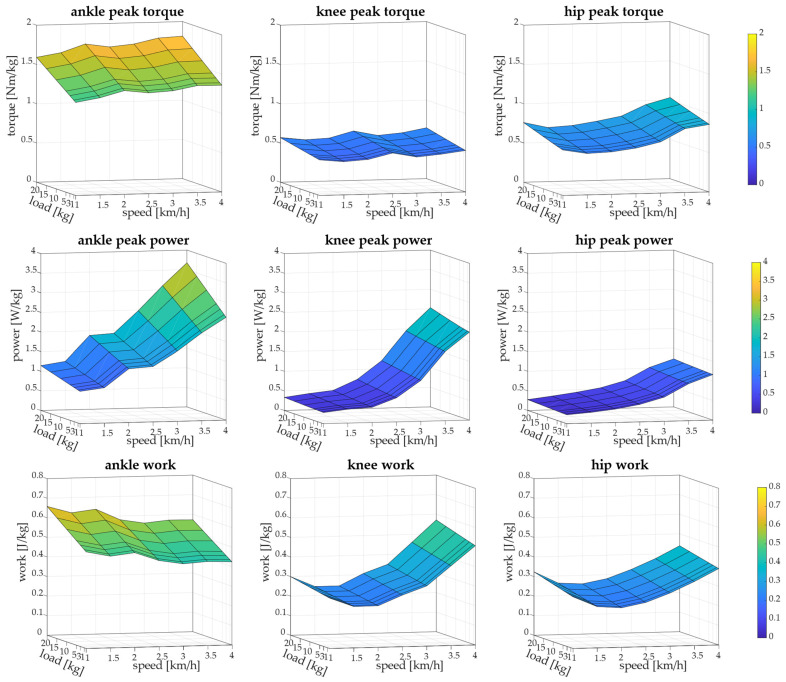
Three-dimensional surfaces show articular biomechanics. Peak torque of ankle, knee, and hip when changing speed and load is reported in first row. Peak power of ankle, knee, and hip when changing speed and load is reported in second row. Work of ankle, knee, and hip when changing speed and load is reported in last row.

In Figure 4, surfaces depicting the variation of peak torque, peak power, and work of all joints when changing speed and load averaged across subjects are reported.

Ankle peak torque increased with both speed and load and, therefore, the minimum was at 1 km/h with load = 0 kg and it was significantly lower than torque at speed > 2 km/h (*p* < 0.006). Statistical tests showed that both speed and load had effects on ankle torque (*p* < 0.001), but no interaction was found. Knee peak torque, instead, slightly changes with load and speed, but both speed and load had effects on knee torque (*p* = 0.002 for speed, *p* = 0.001 for load) with no interaction. Knee peak torque was significantly lower than torque at 2.5 km/h (*p* = 0.03) and 4 km/h (*p* = 0.007). Hip peak torque was affected by speed and load (*p* < 0.001, with no interaction) and was minimum at 1.5 km/h for all loads, and maximum at 4 km/h for all loads. Hip torque at 1.5 km/h was significantly lower than toque at 3 km/h, 3.5 km/h, and 4 km/h (*p* < 0.001) and hip torque was significantly higher with load ≥ 10 kg than with load ≤ 3 kg (*p* = 0.01).

Ankle peak power increased with speed and load (*p* < 0.001) but with no interaction between speed and load. Ankle peak power reached the maximum at 4 km/h with 20 kg. Peak power of the ankle joint was significantly lower at 1 km/h and 1.5 km/h than the other speeds (*p* < 0.001), and power at 2 km/h and 2.5 km/h was lower than speed ≥ 3 km/h (*p* < 0.001). Knee peak power increased with speed (*p* < 0.001). No significant effects of load were found (*p* = 0.1) and no interaction between speed and load. Knee peak power at speed ≤ 2.5 km/h was significantly lower than power at speed ≥ 3 km/h (*p* < 0.001). Hip peak power was affected only by speed (*p* < 0.001) and not by load (*p* = 0.5), and the maximum value was at 4 km/h for all loads, which was significantly higher than power at other speeds (*p* < 0.001). 

Ankle work changed with both speed and load (*p* < 0.001) with no interaction and showed the highest value at 1 km/h with 20 kg. Ankle work decreased with increasing speed. Knee work was affected by speed (*p* < 0.001) and load (*p* = 0.003) and reached the maximum at 4 km/h. Minimum knee work was at 1.5 km/h in all load conditions and work at 2.5 km/h and 2 km/h was significantly lower than the other speeds (*p* < 0.01). Finally, hip work was affected by both speed and load (*p* < 0.001) and was minimum at 2 km/h in all load conditions. Hip work at 2 km/h was significantly lower than work at 1km/h (*p* < 0.001) and at speeds ≥ 3 km/h (*p* < 0.001). In Table 1, Table 2 and Table 3, peak torque, peak power, and work are reported in detail.

## 4. Discussion

### 4.1. The Biomechanical Simulations

In this paper, we simulated different gait conditions with OpenSim. We tested seven gait speeds, ranging from 1 to 4 km/h, and seven simulated carried load conditions, 0 kg, 1 kg, 3 kg, 5 kg, 10 kg, 15 kg, and 20 kg, and all the combinations between the two parameters. Kinematics and dynamics are reported in detail graphically and in tables. Comprehensive data are provided in a wide variety of conditions, also reaching some unusual conditions such as very slow (1 km/h) speed and high loads (20 kg). For this reason, a simulation approach was chosen in order to test numerous conditions that cannot be assessed easily in experimental trials.

The data used in this study came from the dataset of Moreira et al. [14] and our simulations reproduced well the experimental profiles of both kinematics and joint torques and the effects of speed on these parameters. Indeed, the results obtained as maximum torques were in accordance with the experimental data. Similar results in joint torques were found by Fukuchi et al., in which slow, fast, and comfortable gait speed were compared [27]. For the ankle torque, similar changes with speed were found by Palmer, even though only three velocities, namely low, normal, and fast walking, were assessed [15]. Moreover, Lenton et al. showed that, increasing load or speed, the total power and the joint-specific power increase, especially for the knee and hip [18]. 

Results of the effects of load are in agreement with previous studies that showed that the presence of carried loads may increase joint moments to counteract the additional stress and external moments acting on the joints [17]. In particular, the addition of the external load shows two mechanical effects: at the beginning of the stance phase, knee and hip moments increase to accept the increased body weight and at the end of the stance phase, the ankle plantarflexion moment increases to give the propulsion for moving the body forward in the next step [28,29,30]. In our results, hip flexion torque increases more than extensor torque, in line with previous studies [12], but in contrast with other studies, which showed an increase in the hip extensor moment in loaded conditions [31,32]. This may be related to the limited applied load. in fact, significant changes were found for loads greater than 20 kg [28,31]. Moreover, power increases more in the ankle than in the hip when increasing load, agreeing with the shift of the power to the distal joints found in other studies [33,34]. Load carriage was also shown to increase the power of the knee joint [18,35]. 

Both speed and load affect the biomechanics of gait, by scaling the peak values of some of the parameters of the assessment. In particular, load had more effects on ankle torque, hip torque, and hip work, while speed affected hip power and knee work more. Interestingly, we found that ankle work decreases when speed increases, even though ankle power increases. Since the mechanical work is computed as the integral of the power in time, the ankle work decreases because the stride time decreases more than the increased power. This means that the energetic efficiency increases. Indeed, at 4 km/h, gait should be biomechanically more fatiguing than gait at a slower speed but the mechanical work is lower. Interestingly, normal gait speed is usually between 4 km/h and 5 km/h [36], and this means that humans select the gait speed in a range in which the mechanical work required at the ankle level is lower, following the energetic efficiency [37]. Future work, including an even wider range of velocities, will confirm whether there exists a biomechanical optimum and if the OpenSim simulated environment confirms the findings of previous experimental data achieved without the carried load [38]. Moreover, ankle work was the minimum at 3 and 4 km/h for loads lower than 5 kg and at 3 km/h for higher loads. Indeed, experimental assessments showed that humans tend to decrease the gait speed while carrying a load [17]. This could be related to the need to be more energy-efficient. A similar behavior was found in upper limb movements. In fact, the optimal velocity of movement, in which the mechanical work at the shoulder level was the lowest, decreases when increasing the carried load [39].

In the literature, most of the studies analyzed the effects of speed and load on gait biomechanics separately. The speed influence was investigated mainly in clinical scenarios [14,16,27]; the effects of load carriage during gait, instead, were studied in more practical situations, like backpack carriage by schoolchildren or militaries [13,28] and for comparisons between genders [12,17]. Only Lenton et al. [18] analyzed the effects of both speed and load during gait, but in a limited range of values. However, no study analyzed a variety of gait speeds and carried loads, including their effects on lower limbs’ biomechanics. Assessing both speed and load effects can provide a comprehensive characterization of gait biomechanics, and how the two effects combine. Indeed, our results showed that gait speed influences more lower limbs’ biomechanics with respect to load and that carrying a load affects mainly the ankle joint. Therefore, our data expand the previous literature, providing new normative data of kinematics and biomechanics of gait-changing speed and load. 

### 4.2. Application of Simulated Approaches

Simulation approaches with OpenSim are widely used in the literature for many tasks, including a gait analysis [20], pedaling [40], and weight lifting [41]. Indeed, simulations in OpenSim allow us to reproduce real-life scenarios and to analyze kinematics, biomechanical parameters, and muscular activations that may be difficult to measure experimentally. Musculoskeletal models have been used for assessing the difference between walking barefoot or with shoes [42], or for comparing muscle strength between healthy people and patients [21,43]. These models have also been used for postural analyses in working environments [44,45]. In motor control analyses, a large number of muscles that cannot be easily assessed in the real world have been considered to investigate how the number and the choice of muscles can affect the synergy structure [46,47].

Although our data are simulated, they can be useful for applications in which different gait conditions are investigated. A gait analysis can provide crucial information for the determination of the level of functional limitation due to pathology, for the evaluation of the improvements over time and to target the rehabilitation therapy [4]. The assessment of the torques acting on joints can be used in many fields: for the diagnosis and treatment of patients, for the design of gait assistive devices, and for the evaluation of the treatment effects [2]. Changes in speed or in load were shown to also have effects on the motor control in children [48]. Pathological conditions can be assessed with respect to comprehensive data in order to have a detailed assessment aiming at targeting the rehabilitation therapy and evaluate motor recovery [49]. Indeed, patients with neurologic conditions show changes in gait kinematics and biomechanics [50]. Some examples of known effects include patients with multiple sclerosis that reduce hip extension due to an excessive activity of quadriceps muscles and a limited activity of hamstrings [51], and patients who experienced stroke reducing joint torques and powers in gait [52]. Moreover, in sport medicine and orthopedics, orthopedic pathological conditions can be compared to normative data to support clinical decisions and to evaluate the risks of injury and, after surgery, a gait analysis coupled with detailed biomechanics could be used for the evaluation of the outcomes [53]. It might be reasonable to think that OpenSim might be applied to forecast possible surgical outcomes. Another interesting application is the investigation of the effects of the interaction with supporting devices such as a lower limb exoskeleton to assess the impact on gait, as well as the use of specific insoles [54,55]. In sport biomechanics, reference biomechanical data can be used to compare athletes’ performance and to improve their movement efficiency [7].

### 4.3. Limitations and Future Work

In this work, the load was applied as a concentrated vertical force in one point to simulate a backpack carriage. More points of application could be explored in future work, including asymmetrical load carriage. Indeed, among adolescents and children, a vertical load unevenly carried might be a common cause of lower back pain. The biomechanical alterations seen in asymmetrical backpack carriage may put some extra load on the lumbar vertebral joints and it might alter frontal knee biomechanics, contributing to low back pain and pathologies in the knee joint [56]. Moreover, in this study, the kinematics was based on real data without load and thus no effect of load was considered on movement, since real datasets exploring changes both in speed and load were not available. Moreover, multiple factors can affect the kinematics when carrying load, such as anthropometric factors, as reported by Middleton et al. [17]. All these parameters cannot be taken into account at the same time in the simulations and, therefore, experimental data are needed. Since, in the literature, experimental data include changes in velocity or changes in loads separately, a dataset that experimentally assesses the gait speed was considered and the load was considered as affecting only the dynamical parameters. However, in the literature, studies found that the changes in kinematics are proportional to the carried load and in our study, a limited range of loads are considered. Previous studies showed that joint moments are not significantly affected by kinematic profile changes [57], and the effects of loads on kinematic profiles are not significant [18]. However, very high loads could indeed influence angular profiles and affect kinematics, especially the hip angle [58], and, therefore, assessments on real data considering a variety of speeds and loads should be considered in the future. We also scaled the GRFs proportionally with load, and only the vertical and antero-posterior components were scaled, since in the literature the effects of load are mainly studied on the vertical and antero-posterior components. We considered that the medio-lateral force would be marginally affected by load and some studies found no significant changes in the medio-lateral force when carrying a load [31,59]. However, other studies showed that carrying a load may also affect the medio-lateral forces [13,60] and this may affect joint moments, especially hip abduction. Finally, our data are the results of a simulated approach and, therefore, they may be affected by algorithms and optimization pipelines of the musculoskeletal model and may not be completely representative of the reality. 

Despite these limitations, this study expands the present literature, including multiple gait conditions that have not been investigated yet and adding extreme conditions that cannot be easily assessed experimentally, as already conducted in upper limb movements [39]. These results may be useful as normative data in multiple applications, such as clinical, rehabilitative, orthopedics, and industrial. The simulation approach allows us to analyze all the structures and muscles involved in the movement, allowing a complete assessment. Future developments will include the analysis of muscle activations and muscle synergies, allowing us to also assess the motor control strategies at the neural level [61], as already conducted in upper limb movements [46,47]. In this way, a digital twin model could be created in which multiple conditions can be assessed and both biomechanics and motor control can be evaluated, leading to personalized healthcare [62]. For example, in rehabilitation, the model can be used for simulating the ideal condition to improve motor recovery without overloading particular structures and avoiding the risk of injury. The same approach can be used in industrial scenarios in which working postures and the interaction with other devices can be tested to evaluate the risk of musculoskeletal disease. 

## 5. Conclusions

In this work, a musculoskeletal model developed in OpenSim was used to simulate gait when changing speed and carried load, and the effects on biomechanics were investigated. Experimental data from 15 subjects of a publicly available dataset were taken as input to the model. Seven gait speeds and seven carried loads were simulated and torque, power, and mechanical work at ankle, knee, and hip joints were analyzed. All the parameters were significantly affected by speed and almost all measures increased with load. Comprehensive data were provided graphically and with tables, which could be useful for a wide variety of applications, such as rehabilitation, orthopedics, and industrial.

## Figures and Tables

**Table 1:** Means and standard deviations across subjects of peak torques of ankle, knee, and hip when changing speed (in the columns) and load (in the rows). Values are reported in Nm/kg.

Peak Torque [Nm/kg]								
		1 km/h	1.5 km/h	2 km/h	2.5 km/h	3 km/h	3.5 km/h	4 km/h
**ankle**	**0 kg**	1.18 (0.21)	1.24 (0.18)	1.32 (0.16)	1.28 (0.24)	1.30 (0.21)	1.36 (0.24)	1.36 (0.19)
	**1 kg**	1.20 (0.21)	1.26 (0.18)	1.34 (0.16)	1.30 (0.24)	1.32 (0.21)	1.38 (0.24)	1.38 (0.19)
	**3 kg**	1.25 (0.21)	1.30 (0.18)	1.38 (0.16)	1.35 (0.24)	1.37 (0.21)	1.43 (0.24)	1.43 (0.20)
	**5 kg**	1.29 (0.22)	1.34 (0.19)	1.42 (0.16)	1.39 (0.24)	1.41 (0.21)	1.47 (0.24)	1.47 (0.20)
	**10 kg**	1.39 (0.22)	1.44 (0.20)	1.53 (0.16)	1.49 (0.25)	1.52 (0.22)	1.58 (0.25)	1.58 (0.21)
	**15 kg**	1.49 (0.23)	1.54 (0.21)	1.64 (0.17)	1.60 (0.25)	1.62 (0.23)	1.69 (0.26)	1.70 (0.22)
	**20 kg**	1.59 (0.24)	1.64 (0.22)	1.74 (0.17)	1.70 (0.26)	1.73 (0.23)	1.80 (0.27)	1.82 (0.23)
**knee**	**0 kg**	0.46 (0.22)	0.42 (0.19)	0.44 (0.16)	0.52 (0.28)	0.46 (0.18)	0.49 (0.17)	0.53 (0.15)
	**1 kg**	0.46 (0.22)	0.43 (0.20)	0.45 (0.16)	0.53 (0.29)	0.46 (0.18)	0.50 (0.17)	0.54 (0.15)
	**3 kg**	0.47 (0.23)	0.44 (0.20)	0.46 (0.16)	0.54 (0.30)	0.47 (0.19)	0.51 (0.17)	0.55 (0.16)
	**5 kg**	0.48 (0.23)	0.45 (0.20)	0.47 (0.17)	0.55 (0.31)	0.48 (0.19)	0.51 (0.18)	0.56 (0.16)
	**10 kg**	0.51 (0.25)	0.48 (0.21)	0.49 (0.18)	0.58 (0.33)	0.51 (0.20)	0.54 (0.19)	0.59 (0.18)
	**15 kg**	0.54 (0.26)	0.50 (0.22)	0.52 (0.19)	0.60 (0.35)	0.54 (0.22)	0.57 (0.20)	0.62 (0.20)
	**20 kg**	0.57 (0.28)	0.53 (0.23)	0.55 (0.20)	0.63 (0.38)	0.56 (0.23)	0.59 (0.22)	0.65 (0.21)
**hip**	**0 kg**	0.58 (0.13)	0.53 (0.15)	0.54 (0.17)	0.58 (0.14)	0.65 (0.17)	0.81 (0.39)	0.86 (0.21)
	**1 kg**	0.59 (0.13)	0.53 (0.15)	0.55 (0.17)	0.59 (0.14)	0.66 (0.17)	0.82 (0.39)	0.87 (0.21)
	**3 kg**	0.60 (0.13)	0.55 (0.15)	0.57 (0.17)	0.61 (0.14)	0.68 (0.17)	0.83 (0.38)	0.88 (0.21)
	**5 kg**	0.62 (0.13)	0.57 (0.16)	0.58 (0.18)	0.62 (0.14)	0.69 (0.18)	0.85 (0.38)	0.90 (0.22)
	**10 kg**	0.66 (0.13)	0.61 (0.16)	0.62 (0.18)	0.67 (0.15)	0.73 (0.18)	0.88 (0.36)	0.94 (0.23)
	**15 kg**	0.71 (0.14)	0.65 (0.16)	0.67 (0.19)	0.71 (0.15)	0.77 (0.19)	0.91 (0.34)	0.99 (0.24)
	**20 kg**	0.76 (0.14)	0.69 (0.17)	0.71 (0.20)	0.75 (0.16)	0.81 (0.20)	0.95 (0.33)	1.03 (0.25)

**Table 2:** Means and standard deviations across subjects of peak power of ankle, knee, and hip when changing speed (in the columns) and load (in the rows). Values are reported in W/kg.

Peak Power [W/kg]								
		1 km/h	1.5 km/h	2 km/h	2.5 km/h	3 km/h	3.5 km/h	4 km/h
**ankle**	**0 kg**	0.83 (0.21)	0.90 (0.25)	1.37 (0.35)	1.42 (0.38)	1.79 (0.47)	2.24 (0.48)	2.62 (0.64)
	**1 kg**	0.84 (0.21)	0.92 (0.26)	1.40 (0.35)	1.44 (0.39)	1.82 (0.48)	2.28 (0.49)	2.67 (0.66)
	**3 kg**	0.87 (0.22)	0.95 (0.27)	1.45 (0.37)	1.49 (0.40)	1.89 (0.50)	2.37 (0.50)	2.78 (0.68)
	**5 kg**	0.90 (0.23)	0.99 (0.28)	1.50 (0.38)	1.54 (0.41)	1.96 (0.52)	2.46 (0.52)	2.88 (0.70)
	**10 kg**	0.98 (0.25)	1.07 (0.31)	1.63 (0.42)	1.67 (0.44)	2.14 (0.57)	2.67 (0.56)	3.15 (0.76)
	**15 kg**	1.06 (0.27)	1.15 (0.33)	1.75 (0.45)	1.79 (0.47)	2.31 (0.62)	2.89 (0.61)	3.41 (0.83)
	**20 kg**	1.14 (0.30)	1.24 (0.36)	1.88 (0.49)	1.92 (0.50)	2.48 (0.67)	3.11 (0.65)	3.68 (0.89)
**knee**	**0 kg**	0.30 (0.10)	0.36 (0.13)	0.40 (0.10)	0.62 (0.23)	1.04 (0.39)	1.75 (0.48)	2.23 (0.68)
	**1 kg**	0.30 (0.10)	0.37 (0.13)	0.40 (0.10)	0.63 (0.23)	1.05 (0.39)	1.76 (0.49)	2.24 (0.68)
	**3 kg**	0.30 (0.10)	0.37 (0.13)	0.41 (0.11)	0.64 (0.24)	1.07 (0.40)	1.78 (0.50)	2.26 (0.69)
	**5 kg**	0.31 (0.10)	0.38 (0.14)	0.42 (0.11)	0.65 (0.25)	1.08 (0.41)	1.80 (0.51)	2.28 (0.69)
	**10 kg**	0.32 (0.10)	0.39 (0.14)	0.44 (0.11)	0.68 (0.27)	1.12 (0.42)	1.84 (0.54)	2.36 (0.70)
	**15 kg**	0.33 (0.10)	0.41 (0.15)	0.47 (0.12)	0.71 (0.30)	1.16 (0.44)	1.89 (0.57)	2.43 (0.72)
	**20 kg**	0.34 (0.11)	0.42 (0.16)	0.49 (0.13)	0.75 (0.32)	1.21 (0.46)	1.94 (0.60)	2.51 (0.73)
**hip**	**0 kg**	0.24 (0.09)	0.28 (0.16)	0.35 (0.11)	0.45 (0.12)	0.62 (0.17)	0.94 (0.28)	1.16 (0.26)
	**1 kg**	0.24 (0.09)	0.28 (0.16)	0.35 (0.11)	0.45 (0.12)	0.63 (0.17)	0.95 (0.29)	1.16 (0.26)
	**3 kg**	0.24 (0.10)	0.29 (0.16)	0.36 (0.11)	0.46 (0.12)	0.63 (0.17)	0.95 (0.29)	1.17 (0.26)
	**5 kg**	0.25 (0.10)	0.30 (0.16)	0.37 (0.11)	0.47 (0.12)	0.64 (0.17)	0.96 (0.29)	1.17 (0.26)
	**10 kg**	0.26 (0.10)	0.32 (0.17)	0.39 (0.12)	0.49 (0.12)	0.66 (0.17)	0.98 (0.29)	1.19 (0.27)
	**15 kg**	0.27 (0.10)	0.33 (0.17)	0.41 (0.12)	0.51 (0.12)	0.68 (0.17)	1.00 (0.29)	1.20 (0.27)
	**20 kg**	0.28 (0.10)	0.35 (0.17)	0.43 (0.13)	0.53 (0.12)	0.70 (0.17)	1.02 (0.30)	1.22 (0.27)

**Table 3:** Means and standard deviations across subjects of work of ankle, knee, and hip when changing speed (in the columns) and load (in the rows). Values are reported in J/kg.

Work [J/kg]								
		1 km/h	1.5 km/h	2 km/h	2.5 km/h	3 km/h	3.5 km/h	4 km/h
**ankle**	**0 kg**	0.49 (0.13)	0.47 (0.14)	0.48 (0.11)	0.44 (0.12)	0.42 (0.09)	0.43 (0.11)	0.42 (0.10)
	**1 kg**	0.50 (0.13)	0.48 (0.14)	0.49 (0.12)	0.45 (0.13)	0.43 (0.09)	0.44 (0.11)	0.43 (0.10)
	**3 kg**	0.52 (0.13)	0.49 (0.14)	0.51 (0.12)	0.46 (0.13)	0.44 (0.09)	0.45 (0.11)	0.45 (0.10)
	**5 kg**	0.53 (0.14)	0.51 (0.14)	0.52 (0.12)	0.47 (0.13)	0.45 (0.09)	0.46 (0.12)	0.46 (0.11)
	**10 kg**	0.57 (0.15)	0.54 (0.15)	0.56 (0.13)	0.51 (0.14)	0.49 (0.10)	0.50 (0.12)	0.50 (0.11)
	**15 kg**	0.62 (0.16)	0.58 (0.16)	0.60 (0.14)	0.55 (0.15)	0.53 (0.10)	0.54 (0.13)	0.53 (0.12)
	**20 kg**	0.66 (0.17)	0.62 (0.17)	0.64 (0.15)	0.58 (0.16)	0.56 (0.11)	0.57 (0.14)	0.57 (0.13)
**knee**	**0 kg**	0.26 (0.09)	0.21 (0.06)	0.21 (0.05)	0.26 (0.13)	0.31 (0.07)	0.41 (0.07)	0.51 (0.11)
	**1 kg**	0.26 (0.09)	0.21 (0.06)	0.21 (0.05)	0.27 (0.13)	0.31 (0.07)	0.41 (0.07)	0.51 (0.11)
	**3 kg**	0.26 (0.09)	0.22 (0.06)	0.22 (0.05)	0.27 (0.14)	0.31 (0.07)	0.41 (0.08)	0.52 (0.11)
	**5 kg**	0.29 (0.09)	0.22 (0.06)	0.22 (0.06)	0.27 (0.14)	0.32 (0.07)	0.42 (0.08)	0.52 (0.11)
	**10 kg**	0.29 (0.10)	0.23 (0.07)	0.23 (0.06)	0.28 (0.15)	0.33 (0.08)	0.43 (0.08)	0.54 (0.12)
	**15 kg**	0.29 (0.10)	0.24 (0.07)	0.24 (0.06)	0.30 (0.16)	0.34 (0.08)	0.45 (0.09)	0.55 (0.12)
	**20 kg**	0.30 (0.11)	0.25 (0.07)	0.24 (0.06)	0.31 (0.17)	0.35 (0.09)	0.46 (0.09)	0.57 (0.13)
**hip**	**0 kg**	0.26 (0.08)	0.21 (0.06)	0.20 (0.06)	0.23 (0.05)	0.27 (0.06)	0.33 (0.09)	0.39 (0.09)
	**1 kg**	0.27 (0.08)	0.21 (0.06)	0.20 (0.06)	0.23 (0.05)	0.27 (0.06)	0.33 (0.09)	0.39 (0.09)
	**3 kg**	0.27 (0.08)	0.22 (0.06)	0.21 (0.06)	0.23 (0.05)	0.28 (0.06)	0.33 (0.09)	0.40 (0.09)
	**5 kg**	0.28 (0.08)	0.22 (0.06)	0.21 (0.07)	0.24 (0.05)	0.28 (0.06)	0.34 (0.09)	0.40 (0.09)
	**10 kg**	0.29 (0.08)	0.24 (0.06)	0.23 (0.07)	0.25 (0.06)	0.30 (0.07)	0.35 (0.09)	0.41 (0.09)
	**15 kg**	0.31 (0.08)	0.25 (0.06)	0.24 (0.07)	0.27 (0.06)	0.31 (0.07)	0.36 (0.09)	0.43 (0.10)
	**20 kg**	0.32 (0.09)	0.26 (0.07)	0.26 (0.08)	0.28 (0.06)	0.32 (0.07)	0.38 (0.09)	0.44 (0.10)

## Data Availability

The data presented in this study are available on reasonable request from the corresponding author.
